# Kidney protection during surgery on the thoracoabdominal aorta: a systematic review

**DOI:** 10.1093/icvts/ivaf093

**Published:** 2025-04-11

**Authors:** James Thomas Bennett, Sarah Shirley, Patricia Murray, Bettina Wilm, Mark Field

**Affiliations:** Liverpool Heart and Chest Hospital NHS Trust, Liverpool, United Kingdom; Faculty of Health and Life Sciences, The University of Liverpool, Liverpool, United Kingdom; Liverpool Heart and Chest Hospital NHS Trust, Liverpool, United Kingdom; Faculty of Health and Life Sciences, The University of Liverpool, Liverpool, United Kingdom; Faculty of Health and Life Sciences, The University of Liverpool, Liverpool, United Kingdom; Faculty of Health and Life Sciences, The University of Liverpool, Liverpool, United Kingdom; Liverpool Heart and Chest Hospital NHS Trust, Liverpool, United Kingdom; Faculty of Health and Life Sciences, The University of Liverpool, Liverpool, United Kingdom

**Keywords:** thoracoabdominal surgery, aorta, kidney, protection, renal, perfusion

## Abstract

**OBJECTIVES:**

Acute kidney injury (AKI) is a common consequence of surgical repair of the thoraco-abdominal aorta (TAA). Perfusion techniques aim to facilitate renal protection through oxygenation or hypothermia. This systematic review assesses renal and mortality outcomes by perfusion techniques to evaluate their ability to provide effective kidney protection.

**METHODS:**

PubMed, Web of Science, ClinicalTrials.gov and ClinicalTrialsRegister.EU were searched to identify relevant studies published from 1995 to 2024. Following quality assessment and data extraction, outcomes of the highest quality studies were used to synthesize a narrative discussion.

**RESULTS:**

Thirty-eight studies were analysed, featuring three extracorporeal strategies: left heart bypass (LHB; n = 22), cardiopulmonary bypass with deep hypothermic circulatory arrest (DHCA; n = 11) and partial cardiopulmonary bypass (pCPB; n = 10). Three categories of selective renal perfusion (SRP) strategy were identified: warm blood, cold blood and cold crystalloid. Five studies of ‘very high’ and ‘high’ quality demonstrate a 0–13.6% incidence of post-operative dialysis and 5.0–13.3% risk of operative mortality following LHB with cold crystalloid SRP. No studies in support of DHCA or pCPB provided a high quality of evidence.

**CONCLUSIONS:**

Left heart bypass with crystalloid SRP provides a benchmark for rates of dialysis and mortality following TAA repair. However, AKI remains significant, emphasizing the need for continued innovation in SRP, and a greater understanding of overlooked risk factors. DHCA and pCPB are supported by low-quality evidence, meaning that prospective research is necessary to enable fair comparison. Finally, consensus on data reporting is recommended to improve the quality of future studies in this area.

## GRAPHICAL ABSTRACT

**Figure:**
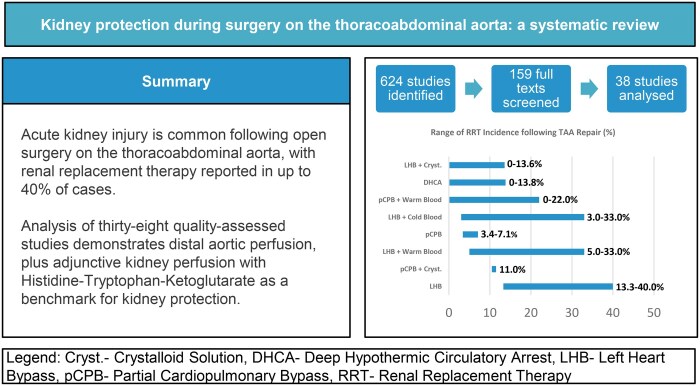

## INTRODUCTION

### Background

Surgical repair of the thoraco-abdominal aorta (TAA) is associated with a high incidence of post-operative acute kidney injury (AKI) and accompanying risks of renal replacement therapy (RRT) and early mortality [1–10]. Whilst intraoperative perfusion techniques may be employed to provide organ protection and improve patient quality of life following surgery [10, 11], attaining effective renal protection remains a challenge during extensive repairs.

**Figure 1:**
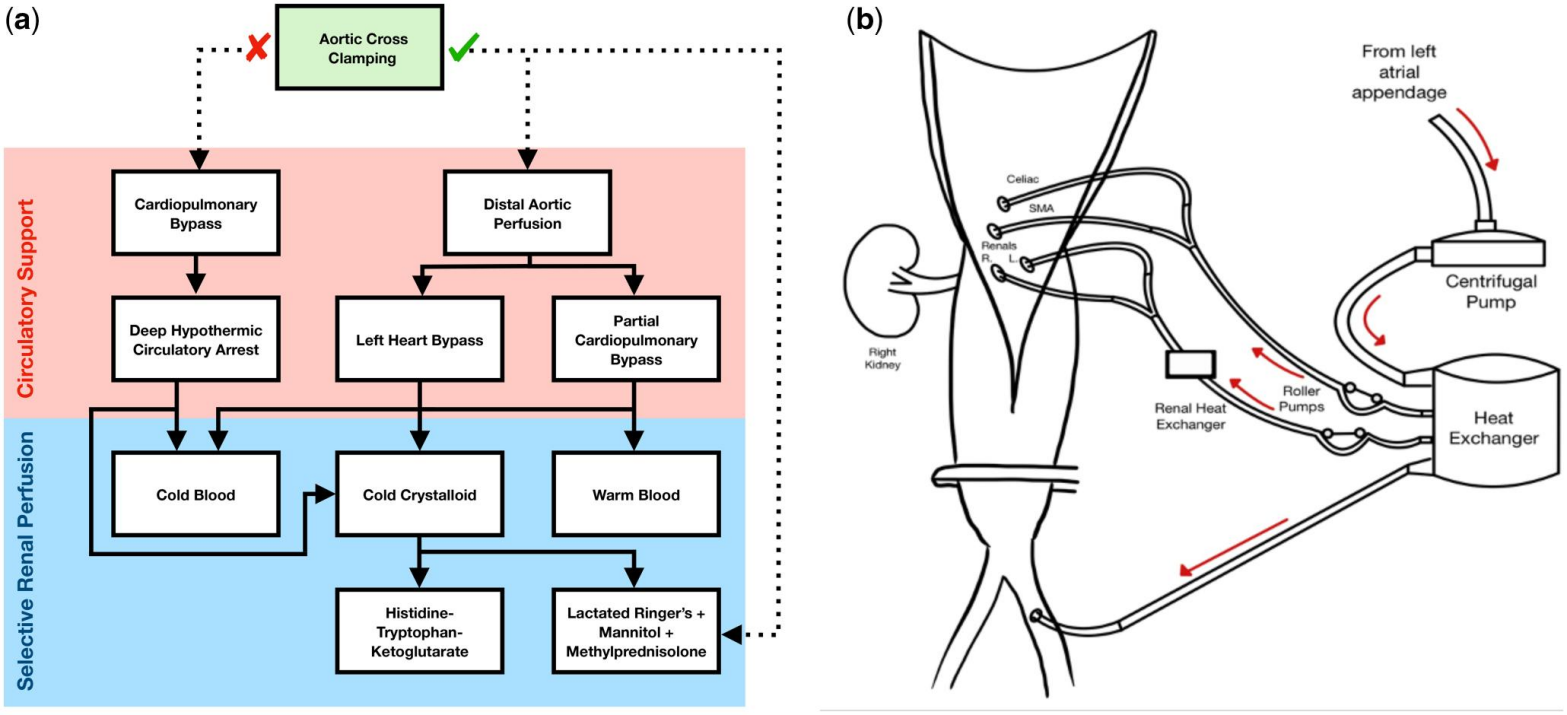
Strategies of circulatory support and selective renal perfusion used during TAA repair. (**a**) Decision tree for the use of different circulatory support and selective renal perfusion strategies. Circulatory support strategies require the use of the heart lung machine for systemic blood flow. Selective renal perfusion involves direct cannulation and perfusion of the renal arteries, and may be used alongside, or independently of, systemic circulatory support. (**b**) Schematic of left heart bypass with adjunctive selective renal blood perfusion. A centrifugal pump is used to shunt oxygenated blood from the left side of the heart to the distal aorta, via a heat exchanger to maintain systemic normothermia or mild hypothermia. Roller pumps can be used to actively pump blood to the visceral arteries. In this example, a renal heat exchanger is used to control the temperature of blood before it is selectively delivered into the renal arteries

Renal ischaemia-reperfusion injury (IRI) plays a key pathophysiological role in the development of AKI, inciting cellular injury through adenosine triphosphate depletion, reactive oxygen species generation and localized inflammation [11–16]. Whilst the practice of ‘simple aortic cross clamping’ (SACC) demands expedient surgery to minimize the duration of renal ischaemia [5, 17, 18], extracorporeal perfusion strategies may attenuate renal IRI through oxygenation or hypothermia (Fig. 1a).

Left heart bypass (LHB) or partial cardiopulmonary bypass (pCPB) can facilitate distal aortic perfusion (DAP) and perfuse the kidneys during surgery (Fig. 1b) [12, 18]. Whilst LHB actively shunts *oxygenated* blood to the distal aorta, pCPB utilizes *venous* blood and requires an oxygenator for gas exchange. Alternatively, full cardiopulmonary bypass with deep hypothermic circulatory arrest (DHCA) can facilitate systemic cryoprotection [19].

All strategies can be supplemented with selective renal perfusion (SRP) (Fig. 1a and b). ‘Warm’ SRP, delivered at normothermia to mild hypothermia, requires blood to maintain oxygen delivery; ‘cold’ SRP, cooled to approximately 4°C, aims to reduce renal oxygen consumption and can utilize blood and/or crystalloid solutions [1, 20].

A 2010 report from the American Heart Association recommends that cold blood or crystalloid kidney perfusion is considered during renal artery exposure [21]. Beyond this, limited guidance exists to inform best practice in renal protection. This systematic review evaluates published renal and mortality outcomes following TAA repair to synthesize a narrative discussion on the quality of findings, and the efficacy of renal protection strategies. Our objectives are as follows:

1. To assess the quality of evidence supporting the use of relevant perfusion techniques.
2. To evaluate the ability of perfusion techniques to ameliorate AKI and operative mortality.
3. To identify important areas of research for future innovation in renal protection.

## MATERIALS AND METHODS

### Protocol and literature search

Our protocol was registered with Prospero (CRD42020166428) and designed according to Preferred reporting items for systematic reviews and meta-analyses (PRISMA) and Synthesis without meta-analysis (SwiM) recommendations. Searches of PubMed, Web of Science, ClinicalTrials.gov and the EU Clinical Trials Register were conducted to identify relevant English language studies published between 1995 and 2024. Studies were screened against eligibility criteria at the abstract and full-text stages. Included studies were required to feature open surgery on the TAA, an adult patient population, the use of extracorporeal circulation and reporting of renal outcomes. Studies containing the predominant use of endovascular techniques or type A dissection repairs were excluded. Clinical trials and observational studies were included, whereas single case studies and non-primary publications were excluded. Full literature search terms are provided in Table S1.

### Data extraction

Data extraction was performed using Covidence, by two reviewers (J.B. and S.S.) to ensure consensus in recorded outcomes. Study characteristics, patient demographics, intervention details and clinical outcomes were extracted. Endpoints included the incidence of post-operative RRT, AKI, 30-day mortality and in-hospital mortality. Where appropriate, patient subgroups were identified within studies by the predominant perfusion strategies used. Data were extracted and presented by patient subgroups, to facilitate the comparison of outcomes by techniques used. Meta-analysis was not undertaken due to the recognized variability in patient characteristics, operative techniques and reported outcomes present in this area of research.

### Outcome measures

#### Acute kidney injury

Post-operative RRT was designated as the primary end-point for AKI, reported in 39/45 (86.6%) studies. Alternative forms of diagnosis, described in 16 (35.5%), were recorded as secondary end-points.

#### Operative mortality

Thirty-day post-operative mortality was reported in 25 of 45 (55.6%) studies, and ‘in-hospital mortality’ in18 (40.0%) studies. Both terms are referred to as ‘operative mortality’ in this review. Two (4.4%) studies did not report mortality.

### Quality and risk of bias assessment

A quality and risk of bias (QROB) assessment was undertaken according to 10 domains:

- Focus on renal protection
- Study design and methodology
- Representativity/homogeneity of baseline patient characteristics
- Risk of participant duplication across multiple studies
- Appropriate statistical analysis
- Certainty of findings, in regard to sample size and magnitude of effect
- Standardization of operative procedures
- Detailed account of perfusion techniques
- Conflicts of interest
- Recruitment timeframe

Studies were judged to present ‘no concerns’, ‘minor concerns’ or ‘major concerns’ for each QROB criterion. For single cohort observational studies, ‘appropriate use of statistical analysis’ was deemed to be ‘not applicable’. The quality of each study was summarized as ‘very high’, ‘high’, ‘moderate’ or ‘low’, based on a scoring system, detailed in Table S2.

Studies with a recruitment timeframe of greater than 5 years were judged to present ‘minor concerns’ and greater than 10 years ‘major concerns’. To account for erroneous findings created by participant duplication, multiple studies conducted at the same institution within an overlapping timeframe were identified and subject to exclusion from systematic analysis. A single paper of the highest quality within each identified group was retained for analysis. Included papers were subsequently judged to present a ‘not applicable’ *risk of participant duplication*. Following QROB assessment, a narrative discussion was synthesized according to the findings of ‘very high’, ‘high’ and ‘moderate’ quality studies.

## RESULTS

### Study characteristics and quality assessment

**Figure 2:**
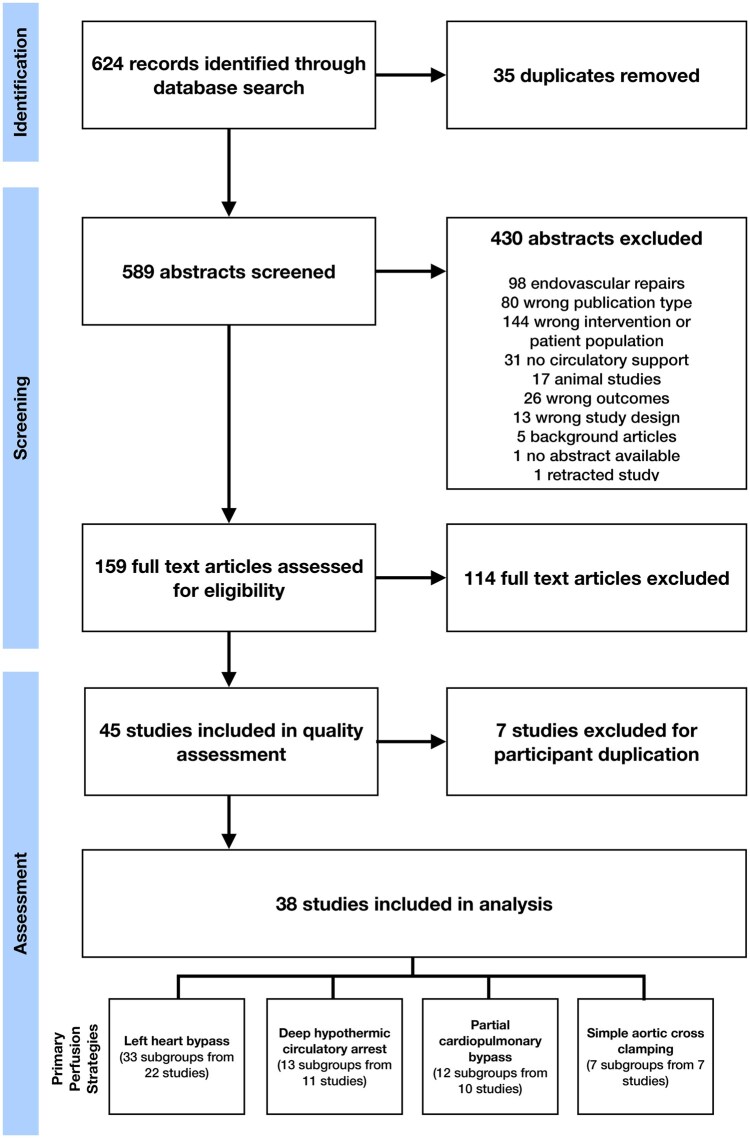
PRISMA flow diagram of the literature search and screening process

**Figure 3:**
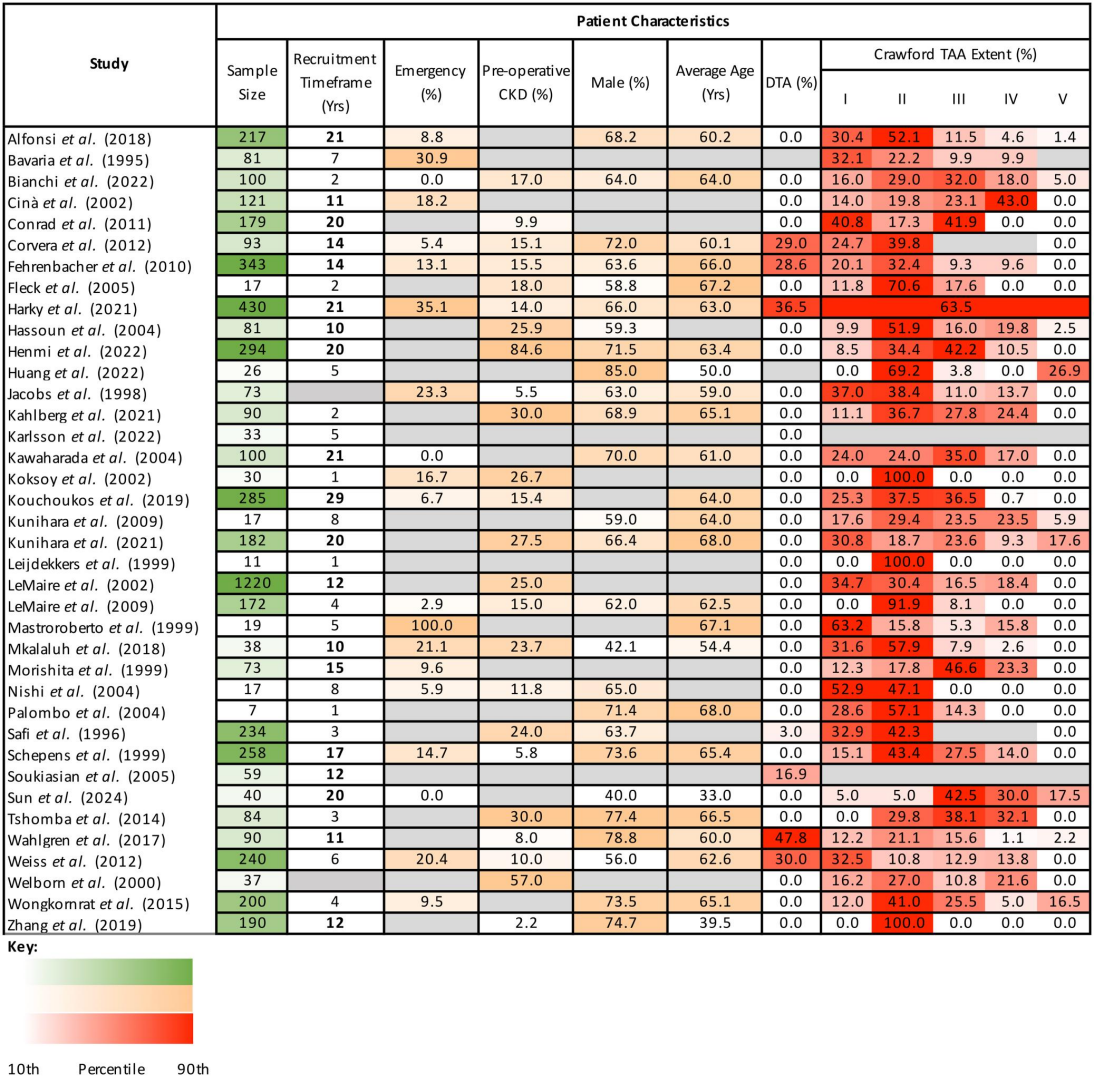
Heatmap of reported patient characteristics from all included studies. The heterogeneity of study characteristics, as illustrated by the variation in sample sizes and proportion of patients with characteristics known to be associated with post-operative renal function. The colour scales used represent 10th to 90th percentiles. CKD: moderate or severe chronic kidney disease; DTA: descending thoracic aorta; TAA: thoracoabdominal aorta; Average age: reported mean or median. Grey cells indicate lack of reporting

**Figure 4:**
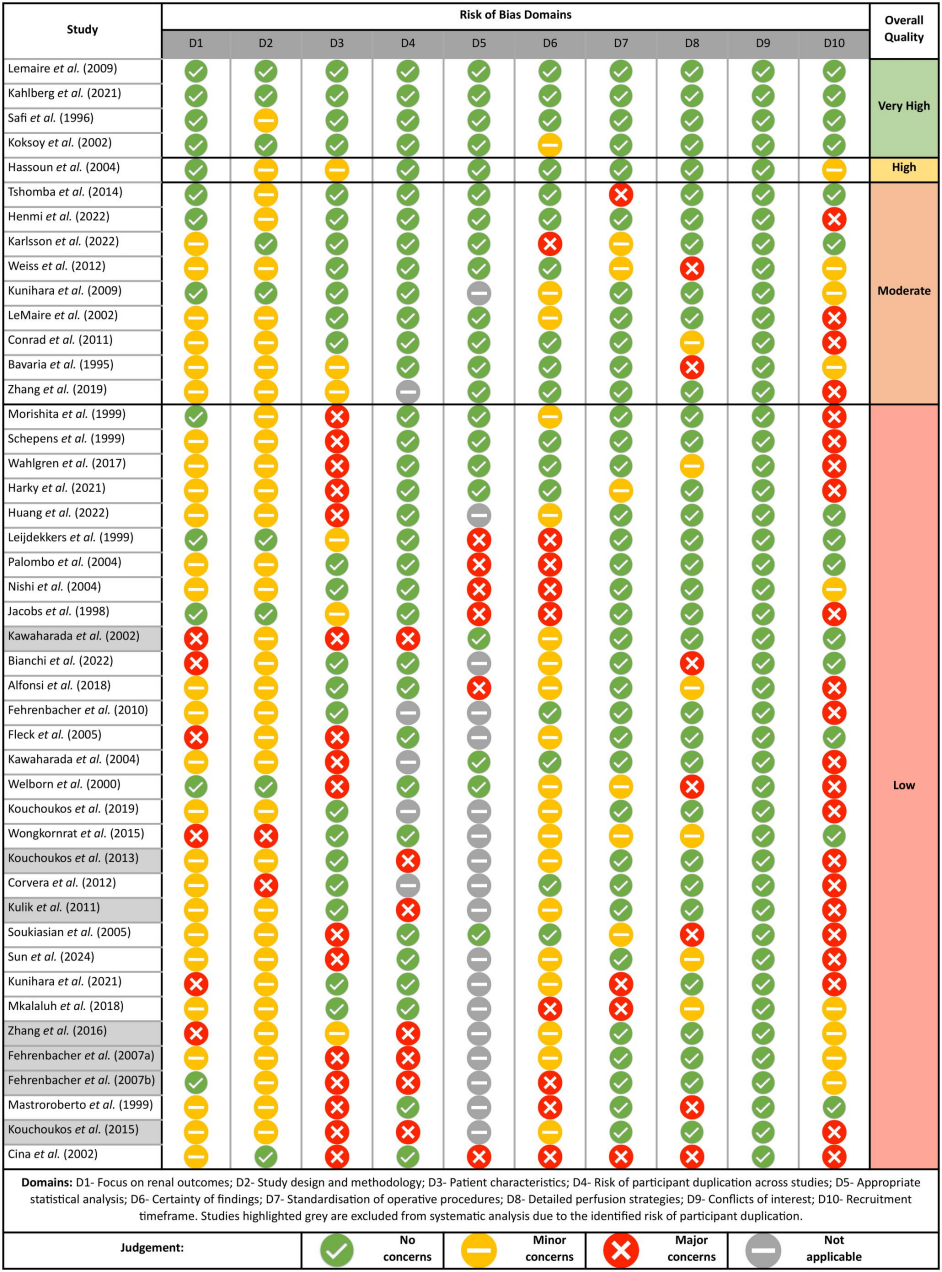
Visualization of QROB assessment for all included studies. Studies were assessed according to 10 risk of bias domains, which were judged to present ‘no concerns’, ‘minor concerns’, ‘major concerns’, or deemed ‘not applicable’. Based on the results to each criterion, studies were scored to present ‘very high’, ‘high’, ‘moderate’ or ‘low’ quality evidence

Forty-five of 589 identified studies met the inclusion criteria (Fig. 2), featuring 3 clinical trials, 7 prospective observational studies (POS) and 35 retrospective observational studies (ROSs). A heatmap of study and participant characteristics is presented in Fig. 3. From the QROB assessment, 4 studies were determined to be ‘very high quality’, 1 ‘high quality’, 9 ‘moderate quality’ and 31 ‘low quality’ (Fig. 4). Seven low-quality studies were subsequently excluded from systematic analysis, due to the identified risk of participant duplication. From the 38 studies that met the requirement for systematic analysis, 61 study subgroups were identified by the perfusion strategies used, featuring 5987 operative cases. Twenty-one studies included multiple patient cohorts, enabling the direct comparison of alternate perfusion strategies, whereas 17 studies featured a single patient group.

### Perfusion techniques

#### Extracorporeal circulation

Three strategies of extracorporeal circulation were identified (Table 1). The exclusive use of LHB was identified in 27 subgroups (17 studies), DHCA in 9 subgroups (7 studies) and pCPB in 10 subgroups (8 studies). Eight subgroups featured mixed perfusion techniques, in which the predominant strategies used were LHB (six subgroups; five studies) and pCPB (two subgroups; two studies). SACC was used exclusively in seven subgroups (seven studies).

**Table 1:** Study characteristics and clinical outcomes

Study	Design	Sample size	Systemic strategy	SRP technique	Extents of surgical repair (%)						AKI endpoint		AKI (%)	Sig.	Mortality endpoint		Mortality (%)	Sig.	Additional evidence of renal protection
					DTA	I	II	III	IV	V	RRT	Other			30 Day	In Hospital			
Bavaria *et al.* (1995) [22]	ROS	45	LHB								**♦**		13.3	NS		**♦**	6.7	<0.05	
		36	SACC										24.2				22.2		
Safi *et al.* (1996) [23]	ROS	90	LHB									**♦**	6.7						**♦**
		58	LHB	Warm blood									29.3						
Jacobs *et al.* (1998) [24]	POS	33	LHB	Warm blood^a^	0	37.0	38.4	11.0	13.7	0	**♦**		9.0			**♦**	8.0		
		40	LHB	Warm blood^b^									5.0						
Leijdekkers *et al.* (1999) [25]	POS	6	LHB	Warm blood	0	0	100	0	0	0	**♦**		33.0						
		5	LHB		0	0	100	0	0	0			40.0						
Mastroroberto *et al.* (1999) [26]	ROS	19	pCPB									**♦**	31.6		**♦**		42.1		
Morishita *et al.* (1999) [27]	ROS	41	pCPB^c^	Warm blood	0	12.2	17.1	53.7	17.1	0		**♦**	4.9	0.01	**♦**		17.0		**♦**
		22	pCPB^d^	Warm blood	0	13.6	27.3	41.0	18.2	0			4.5				5.0		**♦**
		10	SACC		0	10.0	0	30.0	60.0	0			20.0				10.0		
Schepens *et al.* (1999) [28]	ROS	123	SACC	Cold crystalloid	0	13.0	19.5	38.2	29.3	0	**♦**		10.9			**♦**	14.6	0.02	
		135	LHB	Cold crystalloid	0	17.0	65.2	17.8	0	0			5.9				5.9		
Welborn *et al.* (2000) [14]	POS	12	LHB		0	41.7	58.3	0	0	0		**♦**	25.0			**♦**	0.0		
		16	SACC		0	6.3	18.8	25.0	50.0	0			44.0				11.0		
Cina *et al.* (2002) [29]	POS	43	SACC	Cold crystalloid							**♦**		3.0			**♦**	26.6	0.01	
		78	LHB (59%), SACC (41%)	Cold blood									16.0				5.7		
Koksoy *et al.* (2002) [20]	CT	16	LHB	Warm blood	0	0	100	0	0	0	**♦**		6.3	NS		**♦**	6.3		
		14	LHB	Cold crystalloid	0	0	100	0	0	0			0.0				7.1		**♦**
LeMaire *et al.* (2002) [30]	ROS	78	SACC	Cold crystalloid							**♦**		3.0	NS	**♦**		6.0		
		34	LHB	Warm blood									13.0				22.0		**♦**
Hassoun *et al.* (2004) [31]	ROS	44	LHB	Cold blood	0	6.8	47.7	18.2	25.0	2.3	**♦**		23.4		**♦**		21.0	0.02	
		37	LHB	Warm blood	0	13.5	56.8	13.5	13.5	2.7			21.6				46.0		
Kawaharada *et al.* (2004) [32]	ROS	100	pCPB (99%), SACC (1%)	Warm blood	0	24.0	24.0	35.0	17.0	0	**♦**		16.2		**♦**		5.4		
Nishi *et al.* (2004) [33]	ROS	9	pCPB	Cold crystalloid	0	44.4	55.6	0	0	0	**♦**		11.1			**♦**	11.1		
		8	DHCA	Cold crystalloid	0	62.5	37.5	0	0	0			0.0				12.5		
Palombo *et al.* (2004) [34]	ROS	7	pCPB	Warm blood							**♦**		0.0			**♦**	5.9		
Fleck *et al.* (2005) [35]	ROS	17	LHB	Cold crystalloid							**♦**		12.0			**♦**	0.0		
Soukiasian *et al.* (2005) [36]	ROS	39	DHCA								**♦**		0.0	0.04	**♦**		5.0		**♦**
		20	LHB										15.0				20.0		
Kunihara *et al.* (2009) [37]	POS	17	pCPB	Warm blood							**♦**		0.0			**♦**	0.0		
LeMaire *et al.* (2009) [38]	CT	86	LHB	Cold blood	0	0	95.4	4.7	0	0	**♦**		3.0		**♦**		7.0		
		86	LHB	Cold crystalloid	0	0	88.4	11.6	0	0			3.0				5.0		
Fehrenbacher *et al.* (2010) [39]	ROS	343	DHCA		28.6	20.1	32.4	9.3	9.6	0	**♦**		1.5		**♦**		4.4		
Conrad *et al.* (2011) [40]	ROS	52	LHB		0	51.9	17.3	30.8	0	0		**♦**	8.0	NS		**♦**	2.0		
		127	SACC	Cold crystalloid	0	36.2	17.3	46.5	0	0			17.0				5.0		
Corvera *et al.* (2012) [41]	ROS	93	DHCA	Cold blood	29.0	24.7	39.8	[6.45]			**♦**		0.0			**♦**	2.2		
Weiss *et al.* (2012) [42]	ROS	45	DHCA								**♦**		0.0	NS	**♦**		8.9		**♦**
		28	pCPB										7.1				7.1		
Tshomba *et al.* (2014) [43]	ROS	42	LHB (88.9%), SACC (11.1%)	Cold crystalloid	0	0	23.8	45.2	31.0	0	**♦**		2.4		**♦**		9.5		
		42	LHB (84.4%), SACC (15.6%)	Cold HTK	0	0	35.7	31.0	33.3	0			7.2				2.4		**♦**
Wongkornrat *et al.* (2015) [44]	ROS	200	LHB	Cold crystalloid							**♦**		2.5		**♦**		3.0		
Wahlgren *et al.* (2017) [45]	ROS	57	LHB	Cold blood	40.4	12.3	17.5	24.6	1.8	3.5	**♦**		33.0		**♦**		10.5		
		33	DHCA		60.6	12.1	27.3	0	0	0			15.0				6.1		
Alfonsi *et al.* (2018) [46]	ROS	173	LHB	Cold crystalloid							**♦**		4.6		**♦**		5.2		
		41	DHCA										7.3				9.7		
Mkalaluh *et al.* (2018) [47]		38	pCPB (84.2%), DHCA (15.8%)								**♦**		10.5		**♦**		10.5		
Kouchoukos *et al.* (2019) [48]	ROS	285	DHCA								**♦**		6.3		**♦**		7.4		
Zhang *et al.* (2019) [49]	ROS	58	DHCA								**♦**		13.8		**♦**		6.9	<0.05	
		58	pCPB										3.4				0.0		
Harky *et al.* (2021) [50]	ROS	273	LHB, DHCA (ratio not reported)	Cold blood							**♦**		22.7		**♦**		9.9		
Kahlberg *et al.* (2021) [2]	CT	45	LHB	Cold HTK		11.1	37.7	33.3	20.0	0	**♦**		2.3		**♦**		6.7		**♦**
		45	LHB	Cold crystalloid		11.1	35.5	22.2	28.8	0			13.6				13.3		
Kunihara *et al.* (2021) [51]	ROS	182	LHB (86.8%), DHCA (13.2%)								**♦**		12.1		**♦**		11.0		
Bianchi *et al.* (2022) [52]	POS	100	LHB (84%), SACC (16%)		0	16.0	29.0	32.0	18.0	5.0		**♦**	8.0			**♦**	8.0		
Henmi *et al.* (2022) [53]	ROS	281	pCPB (86.5%), DHCA (13.5%)		0	8.9	35.9	44.1	11.0	0	**♦**		12.8			**♦**	8.2		
Huang *et al.* (2022) [54]	ROS	26	LHB	Cold crystalloid/HTK	0	0	69.2	3.9	0	26.9	**♦**		7.7		**♦**		15.4		
Karlsson *et al.* (2022) [55]	POS	21	LHB	Cold blood							**♦**		33.0			**♦**	5.0		
		12	LHB	Cold blood									8.0				0.0		
Sun *et al.* (2024) [56]	ROS	40	pCPB								**♦**		5.0		**♦**		7.5		

a No pressure monitoring.

b SRP line pressure >60mmHg.

c Roller pump.

d Centrifugal pump. AKI: Acute kidney injury; CT: Clinical trial; DHCA: Deep hypothermic circulatory arrest; DTA: Descending thoracic aorta; Extents of repair : Crawford classification system; HTK: Histidine-tryptophan-ketoglutarate; LHB: Left heart bypass; NS: Not significant; pCPB: Partial cardiopulmonary bypass; POS: Prospective observational study; ROS: Retrospective observational study; RRT: Renal replacement therapy; SACC: Simple aortic cross clamping; SRP: Selective renal perfusion. Results in square parentheses represent merged groups.

#### Selective renal perfusion

SRP was employed as an adjunct to LHB in 26 subgroups (22 studies), utilizing cold crystalloid solutions in 11 subgroups (9 studies), warm blood in 7 subgroups (6 studies) and cold blood in 7 subgroups (6 studies) (Table 1). The characteristics of renal perfusion were not clearly defined in one study. SRP was used alongside pCPB in eight subgroups (seven studies), including warm blood in seven subgroups and cold crystalloid in one subgroup. Five subgroups featured SACC with the adjunctive use of cold crystalloid SRP. Of three subgroups that reported the use of SRP with DHCA, two used cold blood and one used a cold crystalloid solution. The contents of renal perfusates are displayed in Table 2.

**Table 2:** Reported flow, pressure and volume characteristics of selective renal perfusion strategies.

Category	Study	Perfusate contents	Intermittent or continuous	Flow	Delivery pressure	Volume	Additional information
Cold crystalloid	**Schepens *et al.* (1999)** [28]	Ringer’s acetate + mannitol	Intermittent	100 ml/min		300 ml boluses every 30 min	
	**Koksoy *et al.* (2002)** [20]	Ringer’s lactate + mannitol + methylprednisolone	Continuous			400-600 ml bolus shared between kidneys. Mean total volume 1046 ml.	Kidneys maintained at 15°C.
	**Fleck *et al.* (2005)** [35]	1 l Ringer’s lactate + 100 ml 20% mannitol + methylprednisolone	Intermittent	150 ml/min		450 ml every 10 min	
	**LeMaire *et al.* (2009)** [38]	Ringer’s lactate + mannitol + methylprednisolone	Intermittent			400-600 ml initial bolus followed by 100-200 ml boluses.	Mean total volume 966.1 ml
	**Conrad *et al.* (2011)** [40]	Ringer’s lactate + mannitol + methylprednisolone	Continuous	Rapid bolus followed by continuous infusion		250 ml bolus, infusion rate undefined	Renal temperature maintained at 25°C
	**Tshomba *et al.* (2014)** [43]	Ringer’s lactate + mannitol + methylprednisolone	Continuous	Rapid bolus followed by 10–20 ml/min infusion		500 ml bolus	Maximum dose 1.5 l
		HTK solution	Continuous	Rapid bolus followed by 10-20 ml/min infusion		500 ml bolus	Maximum dose 1.5 l
	**Alfonsi *et al.* (2018)** [46]	Ringer’s acetate + mannitol + methylprednisolone	Intermittent				Bolus administered every 30 min
	**Kahlberg *et al.* (2021)** [2]	HTK solution	Intermittent			1.5 ml per gram of estimated kidney weight	
	**Huang *et al.* (2022)** [54]	Ringer’s lactate + mannitol + methylprednisolone OR HTK solution	Intermittent	300 ml/min	>200 mmHg	200-400 ml every 6 min	
Cold blood	**Hassoun *et al.* (2004)** [31]	Cold blood	Continuous	300-450 ml/min shared with visceral arteries			Temperature of left kidney monitored and maintained at 15-20°C
	**LeMaire *et al.* (2009)** [38]	Cold blood + mannitol + methylprednisolone	Intermittent	100-150 ml/minute	Mean 171 mmHg	300 ml	Mean total volume 1037 ml
	**Corvera *et al.* (2012)** [41]	Cold blood	Continuous	250-300 ml shared with visceral arteries			
	**Karlsson *et al.* (2022)** [55]	Cold blood	Continuous	100-500 ml/min			
Warm blood	**Safi *et al.* (1996)** [23]	Warm blood	Continuous	250 ml/min shared with visceral arteries			Successful flow indicated by 0.5 ml/min urine output
	**Jacobs *et al.* (1998)** [24]	Warm blood	Continuous	60-210 ml/min	>60 mmHg at catheter tip		Pressure-controlled
		Warm blood	Continuous	60-210 ml/min	Unmonitored		Flow-controlled
	**Leijdekkers *et al.* (1999)** [25]	Warm blood	Continuous	400 ml/min per artery			
	**Morishita *et al.* (1999)** [27]	Warm blood	Continuous	200-300 ml/min per artery			
		Warm blood	Continuous	80-220 ml/min per vessel			
	**Koksoy *et al.* (2002)** [20]	Warm blood	Continuous	400 ml/min shared with visceral arteries			
	**Kunihara *et al.* (2021)** [51]	Warm blood	Continuous	500-800 ml/min shared with visceral arteries			
Blood (undefined temperature)	**Kawaharada *et al.* (2004)** [32]	Blood at undefined temperature	Continuous	155 ml/min per artery			Blood flow considered appropriate when patient’s urine output maintained at 0.5 ml/min
	**Henmi *et al.* (2022)** [53]	Blood at undefined temperature	Continuous	150-200 ml/min			

### Reporting of renal ischaemia and perfusion modalities

Selective renal perfusion is intended to reduce the effects of renal ischaemia. Perfusates can be delivered at a range of flows, pressures and volumes, and in the form of intermittent boluses or continuous infusions. The characteristics of 24 different delivery methods were described in detail in 19 studies (Table 2). Only eight studies reported mean renal ischaemia time, and it was not always clear whether this referred to protected or unprotected ischaemia time. This information can be found in the Supplementary Material.

### Outcomes by perfusion strategy

**Figure 5:**
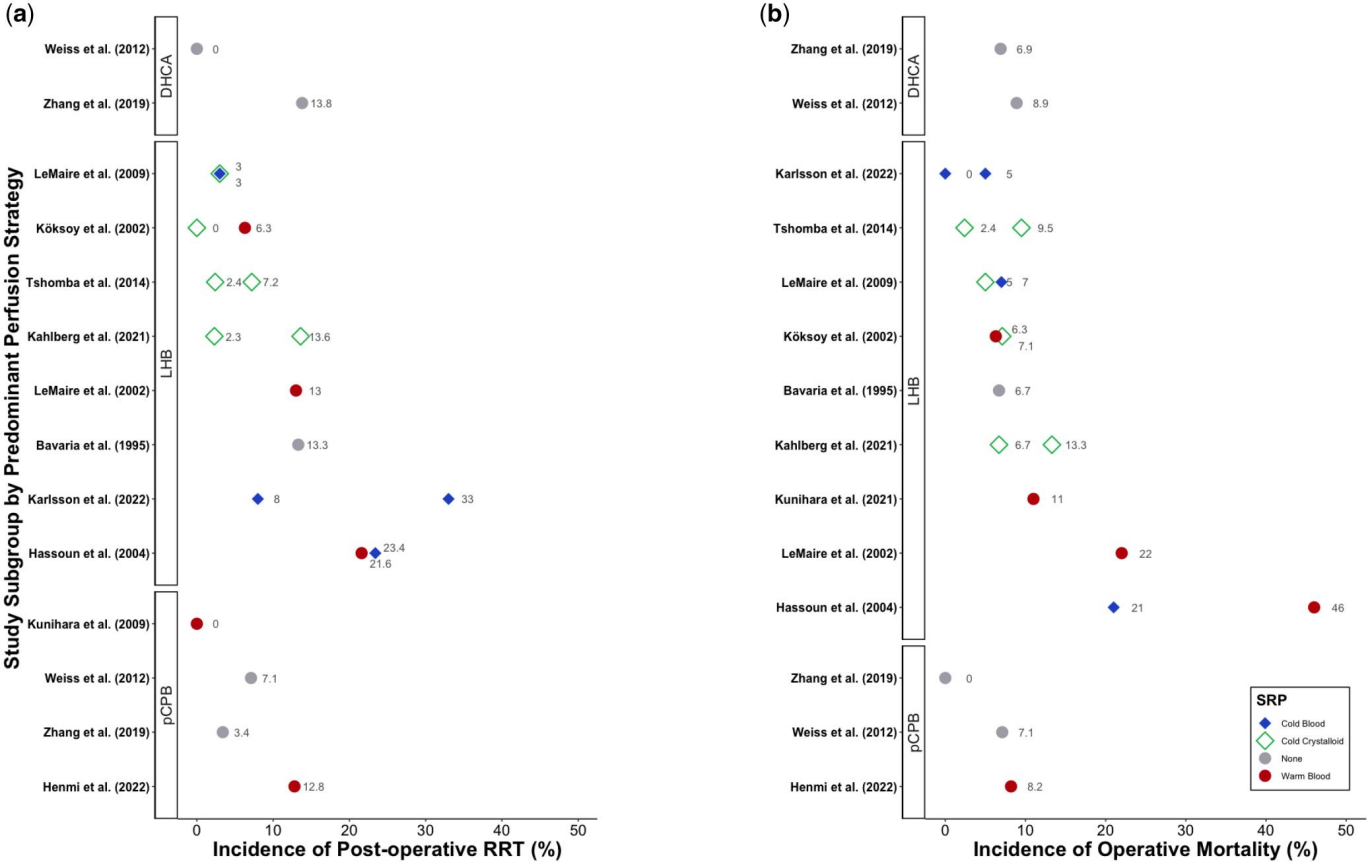
Reported incidences of (**a**) RRT and (**b**) operative mortality for moderate to very high quality studies. Data are presented by study subgroup, categorized by strategy of circulatory support and colour-coded by form of SRP. Studies within circulatory support categories are arranged by mean endpoint incidence. DHCA: cardiopulmonary bypass with deep hypothermic circulatory arrest; LHB: left heart bypass; pCPB: partial cardiopulmonary bypass; SRP: selective renal perfusion

Renal and mortality end-points associated with each study subgroup are presented in Table 1. Outcomes of very-high-, high- and moderate-quality studies are displayed in Fig. 5, where a single circulatory support technique was used for over 75% of patients per subgroup.

#### Distal aortic perfusion: left heart bypass

The use of LHB *without* adjunctive SRP was associated with a 13.3–40.0% incidence of post-operative RRT, and a 0–20.0% risk of operative mortality. When used *with* adjunctive SRP, LHB was associated with incidences of RRT ranging from 5.0 to 33.0% with the use of warm blood, 3.0 to 33.0% with cold blood and 0 to 13.6% with cold crystalloid solutions. Operative mortality ranged from 6.3 to 22.0% with the use of warm blood SRP, 5.7 to 10.5% with cold blood, and 0 to 15.4% with cold crystalloid solutions. The findings of five studies judged to present ‘very-high-’ or ‘high-’quality evidence demonstrate incidences of RRT ranging from 6.3 to 21.6% with warm blood SRP, 3.0 to 23.4% with cold blood SRP and 0 to 13.6% with cold crystalloid SRP.

Four studies demonstrated superior outcomes with LHB, when compared to SACC. Bavaria *et al.* showed that, whilst there was no overall significant difference in the incidence of post-operative RRT, LHB was associated with lower in-hospital mortality (6.7% vs 22.2%, *P* < 0.05) [22]. Schepens *et al.* found that the odds of requiring post-operative RRT were 92% lower with LHB than SACC (OR 0.08, 95%CI 0.01–0.6, *P* = 0.02) [28]. LHB was also associated with a lower incidence of operative mortality (5.9% vs 14.6%, *P* < 0.02) [28]. Notably, a higher proportion of extent II repairs utilized LHB (78.6%), whereas the SACC group included a greater proportion of extent III repairs (66.2%). LeMaire *et al.* demonstrated that LHB could improve rates of AKI compared to SACC (9% vs 32%, *P* < 0.0005), despite supplementing SACC with cold crystalloid SRP. LHB was also associated with non-significant reductions in RRT (3% vs 13%, *P* = 0.167) and 30-day mortality (6% vs 22%) [30]. Similarly, Conrad *et al.* utilized propensity-matching to show that AKI (8% vs 17%, *P* = 0.10) and in-hospital mortality (2% vs 5%, *P* = 0.38) were lower with LHB than SACC with cold crystalloid SRP. However, these outcomes did not reach statistical significance [40]. Together, these findings support the use of DAP for the amelioration of renal IRI.

SRP has been employed alongside LHB in several studies, to varying degrees of success. Warm blood SRP is generally associated with inferior outcomes, whereas selective renal cooling has been shown to provide effective kidney protection (Fig. 5). In 1996, Safi *et al.* compared LHB with warm blood SRP to a non-adjunctive strategy of LHB. They identified warm blood SRP as a risk factor for AKI, associated with a 4-fold increase, independent of TAA extent [23]. The mean collective blood flow was 250 ml/min, shared between the renal and visceral arteries. Similarly, Koksoy *et al.* found that warm blood SRP during extent II repairs was associated with a 3-fold rise in AKI compared to cold crystalloid SRP (63% vs 21%, *P* = 0.03) [20]. Hassoun *et al.* compared warm and cold blood SRP during LHB, discovering that cold blood was associated with a lower incidence of operative mortality (27% vs 56%, *P* < 0.02), yet found no significant difference in recovery from renal dysfunction (27% warm vs 36% cold, *P* = 0.37) [31]. The authors also found that patients who had extent II repair experienced no significant difference in renal recovery. In a 2009 clinical trial, LeMaire *et al.* compared intermittent boluses of cold blood or crystalloid solution during LHB. They found an identical rate of RRT in both cohorts (3.0% vs 3.0%, *P* = 1.0), and no difference in 30-day mortality (7.0% blood vs 5.0% crystalloid, *P* = 0.7) [38]. There was no significantly greater proportion of extent II or III repairs in either patient cohort. Thus, there is strong evidence that renal cooling with blood or crystalloid solutions provides equivalent protection from IRI.

Furthermore, the contents of crystalloid perfusates can affect kidney protection. In a 2021 single-centre clinical trial, Kahlberg *et al.* compared Histidine-Tryptophan-Ketoglutarate (HTK) solution to a Ringer’s-based perfusate, demonstrating a lower incidence of kidney disease improving global outcomes (KDIGO) AKI with HTK (48.9% vs 75.6%, *P* = 0.02), though rates of RRT were lower but not significantly different (2.2% vs 13.3%, *P* = 0.11) [2]. Additionally, no significant difference in 30-day mortality was identified (6.7% HTK vs 13.3% Ringer’s, *P* = 0.23) [2].

#### Distal aortic perfusion: pCPB

pCPB *without* SRP was associated with a 3.4–7.1% incidence of post-operative RRT and a 0–42.1% risk of operative mortality. pCPB with adjunctive warm blood SRP was associated with a 0–16.2% incidence of RRT. However, research into pCPB is limited to older studies with extended recruitment timeframes or small sample sizes.

Yet, like LHB, pCPB has been shown to provide superior renal protection than SACC. Morishita *et al.* demonstrated that pCPB with adjunctive warm blood SRP could significantly reduce the risk of RRT, irrespective of the type of blood pump used for circulatory support (4.5% centrifugal pump, 4.9% roller pump, 20% SACC, *P* = 0.01). pCPB was also associated with higher intraoperative urine production (2.9 ml/h roller SRP, 2.8 ml/h centrifugal SRP, 0.2 ml/h SACC) and lower post-operative serum creatinine concentrations (*P* < 0.0001) than SACC [27]. Whilst this study featured participants recruited between 1982 and 1997, its findings continue to support the value of extracorporeal circulation due to its cohort comparisons. With a small cohort of seventeen patients, Kunihara *et al.* achieved no incidences of RRT or operative mortality with pCPB and warm blood SRP [37]. More recently, Henmi *et al.* analysed 281 cases to show that 150-200 ml/min of warm blood SRP per renal artery was associated with a 12.8% incidence of RRT, a 63.3% incidence of KDIGO AKI and an 8.2% risk of in-hospital mortality [53]. Notably, whilst this study featured a high (84.6%) incidence of pre-operative renal dysfunction and notable proportion of extent II/III repairs (76.6%), recruited participants underwent surgery across a 20-year timeframe.

A propensity-matched study from Weiss *et al.* found no significant difference in the rate of RRT following pCPB or DHCA, though DHCA was associated with a lower incidence of AKI (22.2% vs 46.4%, *P* = 0.03) [42]. Importantly, a higher proportion of DHCA patients received descending thoracic aorta (DTA) repair (22% vs 42%, *P* = 0.04), which is generally associated with reduced renal ischaemic times than TAA repair. Similarly, a propensity-matched study on outcomes of extent II repair, by Zhang *et al.*, showed that whilst pCPB was associated with lower risks of RRT (3.4% vs 13.8%, *P* = 0.047) and 30-day mortality (0% vs 6.9%, *P* < 0.05) than DHCA, there was no significant difference in the rate of new-onset renal insufficiency (13.8% vs 24.1%, *P* = 0.126) [49].

#### Deep hypothermic circulatory arrest

DHCA use is associated with a 0–13.8% incidence of RRT and a 2.2–12.5% risk of operative mortality. Whilst there is some evidence to suggest that DHCA can provide an acceptable standard of renal protection, most studies were judged to present low-quality evidence, and several were removed from systematic analysis due to an identified risk of participant duplication (Fig. 4). As described, Zhang *et al.* demonstrated relatively poor outcomes following DHCA, in comparison to pCPB, though findings were not statistically different [49]. Wahlgren *et al.* compared the use of DHCA with LHB and cold blood SRP, but found no significant differences in rates of AKI or 30-day mortality [45]. However, 47.8% of patients underwent DTA repair, which is typically associated with a lower rate of post-operative renal impairment. Furthermore, whilst there was no significant difference in the proportions of aneurysm extents between patient cohorts, no patients who received DHCA required extent III repair which, in comparison, was undertaken in 25% of LHB cases. Prospective research into the effect of DHCA on kidney protection is warranted, to facilitate the appropriate review of this technique.

## DISCUSSION

Surgical repair of the TAA is a life-saving procedure complicated by high risks of AKI and operative mortality. Addressing these issues requires the use of effective intraoperative kidney protection strategies. This systematic review assesses the outcomes of 38 published studies to identify the incidences of AKI and operative mortality by perfusion techniques. Furthermore, through quality and risk of bias analysis, we evaluate the strength of evidence supporting the use of each technique.

The findings of this review should be interpreted with caution due to the limited number of studies deemed to present high-quality evidence. Just three randomized clinical trials have been conducted in this field, and the majority of studies were judged to be of low quality. Furthermore, study end-points demonstrate a considerable level of variability, due to evolving criteria used in the diagnosis of AKI.

Despite these constraints, several key themes emerged within our analysis of moderate to very-high-quality studies:

1. Distal aortic perfusion can provide effective kidney protection.
2. Adjunctive renal cooling can enhance kidney protection afforded by DAP, during extensive TAA repairs.
3. Warm selective renal perfusion provides inferior kidney protection.
4. Deep hypothermic circulatory arrest requires further investigation.

### DAP provides a benchmark for kidney protection

Normothermic to mild hypothermic perfusion of the distal aorta provides superior kidney protection than SACC. LHB and pCPB can both enable DAP, perfuse the kidneys during suprarenal aortic clamping and reduce their exposure to IRI. During DTA or extent I TAA repairs, DAP can maintain kidney perfusion throughout surgery, as clamping of the infrarenal aorta is not required. During the repair of Crawford extent II-IV aneurysms, however, infra-renal clamping is necessary and renal IRI is unlikely to be avoided. In such cases, sequential aortic clamping may minimize renal ischaemia [5]. The use of LHB is supported by high-quality research, whereas pCPB is supported only by moderate evidence. No studies directly compared the use of LHB to pCPB, and further research into the relative efficacy of both strategies is recommended.

### Renal hypothermia can enhance kidney protection

When DAP cannot facilitate kidney perfusion, the renal arteries may be directly perfused with blood or crystalloid solutions. Our findings demonstrate that cold blood and crystalloid solutions can deliver equivalent, improved protection from AKI and operative mortality [38]. Moreover, HTK solution can further improve renal protection and is linked to a low incidence of post-operative RRT in two studies from one institution [2, 43]. Such strategies should be used with keen awareness of the potential risk of cardiac arrest associated with systemic hypothermia, haemodilution and hyponatraemia. Finally, cold fluids also expose the kidneys to a sudden warm, highly oxygenated environment on reperfusion, which is theorized to exacerbate renal injury [57]. Whilst cold SRP can therefore be used to improve renal protection and reduce the incidence of AKI following surgery, it should be used with caution. There is currently no professional guidance on the management of HTK solution for kidney protection, and further multicentre research into its safe and effective use is advised.

### Warm blood renal perfusion provides inadequate kidney protection

The theoretical advantages of normothermic blood SRP include functional protection of the kidneys and a reduced risk of cardiac arrest. However, several studies featured in our analysis demonstrate that its use is associated with inadequate renal protection and increased mortality [20, 23, 31]. Whilst warm blood SRP during pCPB may provide superior kidney protection than SACC [27], a majority of patients still develop KDIGO AKI [53].

It is feasible that historic applications of warm SRP were performed sub-optimally. The kidneys are metabolically active and typically receive a combined blood flow of 1200 ml/min in normal physiology [58]. Within our included studies, the maximum reported flow provided to the kidneys was 300 ml/min per artery [20]. Furthermore, studies typically employed a branched catheter system to enable simultaneous perfusion of the superior mesenteric and coeliac arteries, which could lead to preferential flow away from the kidneys due to high relative resistance in the renal arteries or cannulae. It is likely that flow rates used in these studies did not provide sufficient oxygen delivery to sustain renal metabolism. Within the related discipline of *ex vivo* renal perfusion, Weissenbacher *et al.* achieved functional normothermic perfusion of human kidneys for 24 hours with a mean flow of 364 ml/min per kidney [59]. This represents a higher flow than used in any studies in this review. Whilst the clinical impact of normothermic perfusion cannot be determined from the results of Weissenbacher’s study, the findings demonstrate that adequate blood flow can sustain functional protection. However, warm perfusion will offer no protection to kidneys during periods of total ischaemia, unlike cold preservation solutions. Without further pre-clinical optimization, we cannot support the use of warm SRP for kidney protection.

### DHCA requires further clinical investigation

Deep hypothermia can reduce global metabolic demand and enable arrest of the systemic circulation. Several studies demonstrate incidences of AKI comparable to the use of LHB with cold crystalloid SRP, suggesting DHCA as an encouraging technique for kidney protection. However, evidence to support the use of DHCA is restricted to low-quality, ROSs. Furthermore, additional clinical outcomes should be considered in the evaluation of novel care practices, and the use of DHCA is reportedly linked to higher rates of post-operative bleeding, stroke, low cardiac output syndrome and prolonged ventilator support than normothermic DAP [11, 60]. Moreover, in our reviewed series, DHCA was the only circulatory support strategy unable to achieve a 0% incidence of operative mortality. Prospective research into the use of DHCA is recommended in order to determine the full implications of its use.

### Emerging risk factors

Evidently, the use of any perfusion strategy is limited in its ability to protect patients from AKI. Whilst clamp-induced renal IRI is understood to be the primary cause, additional risk factors can contribute to its onset and should be considered in any comprehensive renal care strategy. High concentrations of circulating hemeproteins, such as haemoglobin and myoglobin, have been shown to be associated with impaired post-operative renal function.

In 2022, Kiser *et al.* identified intensive use of cell salvage during LHB as a risk factor for AKI and operative mortality [61]. The common strategy of reinfusing unwashed, salvaged blood into the patient circulation is a likely cause of significant intravascular haemolysis. Cardiotomy suction causes red blood cell damage and the release of free haemoglobin into plasma. Elevated free haemoglobin concentration has been associated with AKI following TAA repair and Extra-Corporeal Membrane Oxygenation (ECMO) [62, 63]. Plasma free haemoglobin can scavenge nitric oxide present within the renal vascular endothelium, inhibiting vasodilation to produce renal hypoperfusion. Furthermore, heme, a product of haemoglobin oxidation, has been shown to cause direct proximal tubular cell toxicity, and can trigger an inflammatory cascade leading to cell damage [15, 64]. Haemolysis may therefore contribute to AKI during TAA surgery. Similarly, myoglobinaemia, caused by ischaemia of the femoral arteries, may play a role [65, 66]. The integration of perfusion techniques that minimize red cell damage and peripheral ischaemia are therefore key to reducing renal hypoxia and toxicity.

### Consensus on outcome reporting

The themes within this review are directed by the findings of five studies deemed to present ‘very-high’ or ‘high-quality’ evidence and discussed in relation to the findings of ‘moderate’ studies. Unfortunately, 31 out of 38 included papers were judged to present a low standard of evidence. Major concerns were identified in numerous papers, including recruitment timeframes greater than 10 years (22), skewed or unrepresentative patient cohorts (16), participant duplication across multiple studies (12), lack of certainty on reported outcomes for example due to low sample size (9), insufficient detail on perfusion methods (7), lack of statistical analysis (6), insufficient detail on operative procedures (4) and poor study design or methodology (2). We therefore recommend that international consensus on outcome reporting should be established within the surgical research community to enhance the general quality of research and facilitate innovations in patient care.

### Limitations

This review provides a comprehensive analysis of published studies on renal protection during TAA surgery; however, its findings should be interpreted within the context of its inherent limitations. For example, results are limited to a crude analysis of short-term outcomes, subject to variations in patient characteristics, quality of reported details and AKI diagnosis criteria. Furthermore, we do not analyse certain surgical practices that could influence renal outcomes, such as patch vs Coselli repairs or the use of sequential clamping, renal stenting or endarterectomy. Nor do we include an analysis of renal ischaemic times, due to a paucity in robust data reporting. However, we have included extracted information within our Supplementary Material. Finally, whilst clinical outcomes were independently extracted and judged for consensus by two reviewers, screening and quality assessment was performed by a single reviewer who adhered to a pre-determined set of questions (J.B.).

## CONCLUSION

AKI is common following surgery to repair the thoraco-abdominal aorta and may develop as a result of clamp-induced renal IRI, intravascular haemolysis and myoglobinaemia. The quality of published literature in this area is generally poor, and cohort heterogeneity complicates the task of drawing conclusions on the true efficacy of renal protection strategies. High-quality, prospective research is urgently needed to compare competing strategies of extracorporeal circulation. Moreover, we recommend concerted alignment within the surgical research community on the reporting of patient characteristics, interventions and outcomes. Whilst a small number of high-quality studies establish distal aortic perfusion and adjunctive cold crystalloid SRP as a benchmark for renal protection, rates of AKI remain high. Innovations in SRP techniques and the study of emerging risk factors are required to fully address the problems of AKI and operative mortality.

## Supplementary Material

ivaf093_Supplementary_Data

## Data Availability

The data underlying this article will be shared on reasonable request to the corresponding author.

## Notes

To be submitted to EuroPerfusion 2025 and the Annual Congress of the Society of Clinical Perfusionists of GB&I 2025.

## ABBREVIATIONS

AKI: Acute kidney injury
AKIN: Acute kidney injury network
ATP: Adenosine triphosphate
DAP: Distal aortic perfusion
DHCA: Deep hypothermic circulatory arrest
DTA: Descending thoracic aorta
HTK: Histidine-tryptophan-ketoglutarate solution
IRI: Ischaemia-reperfusion injury
KDIGO: Kidney disease improving global outcomes
LHB: Left heart bypass
pCPB: Partial cardiopulmonary bypass
POS: Prospective observational study
PRISMA: Preferred reporting items for systematic reviews and meta-analyses
QOL: Quality of life
RCT: Randomised controlled trial
ROB: Risk of bias
ROS: Retrospective observational study
RRT: Renal replacement therapies
SACC: Simple aortic cross clamping
SRP: Selective renal perfusion
SwiM: Synthesis without meta-analysis
TAA: Thoracoabdominal aorta
