# Corrigendum to: Kidney protection during surgery on the thoracoabdominal aorta: a systematic review

**DOI:** 10.1093/icvts/ivag199

**Published:** 2026-07-29

This is a corrigendum to: James Thomas Bennett, Sarah Shirley, Patricia Murray, Bettina Wilm, Mark Field, Kidney protection during surgery on the thoracoabdominal aorta: a systematic review, *Interdisciplinary CardioVascular and Thoracic Surgery*, Volume 40, Issue 4, April 2025, ivaf093, https://doi.org/10.1093/icvts/ivaf093

The reference numbers in Tables 1 and 2 are errored and out of sequence from reference 28 in the originally published **REFERENCES** list.

The corrected order to cross reference with the numbers given in the tables should read:
