# Supplementary material for: Kidney protection during surgery on the thoracoabdominal aorta: a systematic review
Source: Interdiscip Cardiovasc Thorac Surg. 2025 Apr 11;40(4):ivaf093. doi: 10.1093/icvts/ivaf093 (PMC12034379; doi:10.1093/icvts/ivaf093)
Supplement: ivaf093_Supplementary_Data [file ivaf093_supplementary_data.zip › CORRECT Supplementary Material.docx]

| **Table s1:** Full Literature Search Terms | |
| --- | --- |
| Information Sources | PubMed  Web of Science  ClinicalTrials.gov  EU Clinical Trials Register |
|  |  |
| Date range searched | 01/01/1995 to 28/06/2024 |
|  |  |
| Types of study to be included | Clinical Trial reports, prospective observational studies and retrospective observational studies. |
|  |  |
| Database Search Queries | **PubMed:**  ((((Thoracoabdominal) OR (TAAA)) AND ((Perfusion) OR (CPB) OR (Bypass)) AND ((Renal) OR (Kidney) OR (AKI) OR (Haemodialysis) OR (Dialysis) OR (Creatinine) OR (GFR) OR (eGFR) OR (Glomerular))) NOT ((Endovascular Techniques[MeSH Terms]))  **Clinicaltrials.gov:**  Condition or disease: Thoracoabdominal Aortic Aneurysm  Other Terms: (Renal) AND (Repair OR Surgery) NOT (Endovascular OR Stent)  Status: Completed OR Unknown status  Age Group: Adult (18-64) OR Older adult (65+)  Sex: All  **EU Clinical Trials Register:**  Search Terms: Thoracoabdominal AND Renal NOT (Endovascular OR Stent)  Age Range: Adult  Trial Status: Completed  **Web of Science:**  (((ALL=(Thoracoabdominal OR TAAA)) AND ALL=(Perfusion OR CPB OR Bypass)) AND ALL=(Renal OR Kidney OR AKI OR Haemodialysis OR dialysis OR Creatinine OR GFR OR glomerular)) NOT TS=(Endovascular Techniques) |

| **Table s2:** Grading criteria used in quality and risk of bias analysis | | |
| --- | --- | --- |
| **Overall quality** | **Number of minor concerns** | **Number of major or not applicable concerns** |
| Very High | ≤1 | 0 |
|  |  |  |
| High | ≤3 | 0 |
|  |  |  |
| Moderate | Any number | 1 |
|  |  |  |
| Low | Any number | ≥2 |
